# Elderly Users’ Emotional and Behavioral Responses to Self-Service Technology in Fast-Food Restaurants

**DOI:** 10.3390/bs13040284

**Published:** 2023-03-26

**Authors:** Jinyoung Nam, Seongcheol Kim, Yoonhyuk Jung

**Affiliations:** School of Media and Communication, Korea University, Seoul 02841, Republic of Korea

**Keywords:** self-service technology, restaurants, elderly users, negative emotions, coping behavior

## Abstract

While COVID-19 has accelerated digital transformation, increasing labor costs and 52-h workweek rules are replacing human labor with self-service technologies (SSTs). Self-service technology is increasingly being implemented in restaurant settings. However, the elderly, who have relatively lower levels of digital literacy, are being excluded from services that can alleviate the economic and social difficulties of their daily lives. This study thus aims to explain how elderly users feel about and respond to SST in fast-food restaurants. An off-site survey was conducted with individuals who had experience using SST. We analyzed the data using the partial least squares structural equation modeling method by SmartPLS 3.0. The results showed that SST’s reduction, perceived ease of use of SST, and perceived time pressure significantly influenced users’ negative emotions toward the SST. However, perceived physical condition and perceived crowding did not have significant influences on users’ emotions. In empirically investigating individuals’ negative emotions toward and coping strategies for challenges posed by SST, this study emphasizes the development of a nationwide digital inclusion policy that can help bridge the digital divide.

## 1. Introduction

Advancements in information and communication technology (ICT) have enhanced service models by transforming interactions between service firms and customers. COVID-19 has accelerated digital transformation, changing service models in various industries. Specifically, changes in the labor environment, such as increased labor costs [1] and 52-h workweek rules, are prompting the replacement of human labor with self-service technologies (SSTs). Many service providers have introduced SSTs to deliver accessible services to consumers, improving efficiency and customer satisfaction [2]. SSTs have been implemented in various service sectors, such as banks, civil services, hospitals, transport terminals, and restaurants. In particular, it is necessary to examine the use of SSTs in the restaurant sector, which is closely tied to individuals’ everyday lives.

Meanwhile, South Korea has experienced a rapid demographic shift with its aging population. By 2040, individuals over 65 years old will account for 33% of the population [3]. Older people with relatively lower levels of digital literacy and deteriorating cognitive and physical abilities are vulnerable to challenges posed by digital technology [4]. Elderly people’s digital information usage was approximately 71.4% in 2020, indicating a relatively lower level of digital skills among this demographic [5]. In particular, SSTs are increasingly being implemented and are expected to become a mainstay in restaurant settings in the near future [6]. The prevalence of self-service kiosks intensifies the digital divide, which refers to the inequalities that individuals experience in terms of access to ICT [7], aggravating the economic and social difficulties faced by elderly people. Older users have lower intentions to adopt SST considering their physical capabilities and barriers to use [8,9]. Technology has become important in everyday life, yet it is too complex, especially for older users [10]. This is one of the most prominent obstacles for older people, drawing attention from both scholars and practitioners. However, there has been scant research exploring elderly users’ actual emotional and behavioral responses to SST use. This study employs coping behavior to explore the relationships among personal, technical, and situational characteristics that lead to older people’s negative emotions toward SST and accompanying coping behaviors. In particular, this study explores older people’s responses to restaurant self-service kiosks, which have already been implemented widely.

## 2. Research Background

### 2.1. SST in South Korea

SST refers to the “technological interfaces that enable customers to produce a service independent of direct service employee involvement” [11]. SST is a service provided by customers in a service encounter using any technological apparatus without the need for direct contact with employees. SST involves customers’ interaction with touchscreen technologies that are prevalent in public (e.g., vending machines, self-scan supermarket checkout machines, ATMs), work (e.g., production control), and individual applications (e.g., smart devices; [12]. SSTs currently operate in various sectors, including restaurants, retail, banking, healthcare, hospitality, education, and government [13].

The global SST market totaled USD 33.66 billion in 2020 and is projected to reach USD 62.39 billion by 2028. Following the increase in market share in South Korea, the domestic kiosk market is expected to increase at an average annual rate of 61.5%, reaching USD 17.6 million in 2020 [14]. The shipment volume of kiosks from domestic manufacturers is estimated to be 12,915 units. COVID-19 and the rapidly increasing minimum wage in South Korea have accelerated SST use in the service industry, including restaurants, convenience stores, and coffee shops. For example, as of 2019, there were kiosks in over 60% of McDonald’s, Lotteria, and Burger King locations [14]. Specifically, kiosk adoption has reached over 70% in McDonald’s stores and over 98% in KFC stores as of 2020.

### 2.2. SST Literature

Prior research has focused on the elderly population’s use of digital technology from an aging perspective [15], a health and psychological perspective [16], a capabilities approach [17], or a social policy perspective [18]. Prior research has examined elderly users’ adoption or usage of general technology, including digital technology, ICT, or mobile banking [19,20,21,22]. There are variations between different groups of elderly users, with lower socioeconomic groups being most likely to become excluded from the benefits of technology [23]. Prior research has analyzed the differences among the older population in their ability to use digital technology, the Internet, and telehealth [24,25,26]. Some studies, therefore, have focused on the differences among older adults’ use of technology, suggesting that their adoption of technology is heterogeneous [27].

In terms of elderly users’ responses to technology, prior studies examined users’ adoption of digital technology [21,28,29] and barriers that restrict them from using technology [30,31]. In terms of SST usage in general, a study investigated the association between servicescape and intentions to use SST [32], while another study examined how consumers’ perceptions of SSTs’ quality affect their emotional and cognitive states and their responses [33]. Concerning SST use by older people, one study examined users’ engagement in the production and consumption of SST from a service management perspective [34]. Additionally, a study examined the intentions to adopt SST among older consumers, considering chronological and subjective age and the future time perspective [9]. Another study investigated older consumers’ determinants of self-service kiosk adoption in fast-food restaurants [35,36] or in self-checkouts [37]. Therefore, prior studies have focused on users’ adoption of technology, differences within the older population’s use of technology, and users’ responses to the use of technology in terms of adoption or barriers. However, there has been scant research examining the behavioral responses of elderly users on the negative aspects of SST, especially the potential challenges it poses for older people. There has been little research examining older people’s actual responses to using SST. To address this research gap, this study specifically examines the relationship between personal, technical, and situational characteristics and older people’s emotional and behavioral responses to SST.

### 2.3. Negative Emotions: Social Anxiety and Helplessness

According to the cognitive appraisal perspective, emotions refer to the mental states of readiness triggered by the assessment of a situation [38]. Technology use triggers emotions and consequently influences users’ attitudes, beliefs, and intentions [39]. Negative emotions can be categorized into anxiety, sadness, anger, and guilt, which are reactions to actual (appraised) or possible harm [40]. The core relational theme for anxiety involves a certain level of threat, and the primary assessments of motivational relevance and incongruence are related to secondary assessments of low emotion-based coping ability. Unlike anxiety, the core theme related to sadness is a loss of helplessness, the inability to restore what was lost, which is related to negative future expectations.

As elderly customers try out SST, they may face difficulties in the process, leading to negative emotions. Negative experiences with SST may trigger emotional responses of social anxiety and helplessness insofar as they evoke a certain level of threat or irrevocable loss. Social anxiety refers to distress related to the awareness of others’ perceptions of oneself as a social object [41]. On the other hand, helplessness is a negative emotional state that arises when frustration develops, such that, despite desiring a change, individuals feel unsure about whether their actions will make a difference [42].

### 2.4. Coping Behavior

Coping behavior is defined as the process of taking measures to reduce stressful situations [43]. Coping indicates individuals’ cognitive and behavioral attempts to deal with external and internal demands [44]. Coping is often prompted by negative emotions, as individuals try to lessen their emotional stress and seek favorable feelings. In this study, we classify coping strategies as problem-based or emotion-based in examining older people’s use of SST. Problem-based coping behavior focuses on resolving and altering the matter to overcome stress [43]. This coping strategy addresses the cause of displacement, alters the environment, or changes the individual in terms of willingness to learn new skills. On the other hand, emotion-based coping attempts to modify individual perceptions and reduce distressing emotions [43]. Therefore, elderly users who face difficulties when using SST may engage in relevant coping strategies after experiencing negative emotions. As a type of problem-based coping behavior, elderly users may seek interpersonal contact and assistance from human employees [45]. Moreover, elderly users may imitate or observe others and learn how to utilize SST [46]. On the other hand, in terms of emotion-based coping, elderly users may behaviorally disengage from the stressor, the SST [47]. Thus, this study employed three specific coping strategies in the context of SST: seeking interaction, observing others, and behavioral disengagement.

### 2.5. SST-Associated Coping Behaviors Mediated by Negative Emotions

Emotions influence individuals’ behavioral responses, as emotions play an adaptive role that is associated with behaviors [48]. Previous research has examined negative emotions as determinants of people’s coping processes [49]. Negative emotions evoke attempts to cope, and these coping strategies may moderate the impact of negative emotion on performance [50].

The negative emotion of social anxiety refers to “the discomfort that is connected with the awareness of other people’s evaluation of oneself as a social object” [41]. Users may become socially anxious if other people observe them using the SST [51]. Correspondingly, individuals who experience anxiety perform relevant adaptive behaviors to reduce the situation’s risk level. In this regard, to reduce social anxiety, elderly users are likely to seek assistance from human employees. Moreover, social learning theory suggests that users’ actions can be affected by observing others’ actions or seeking others’ opinions [46]. In this context, older people may feel the need to control their anxiety about unfavorable situations by observing others using SST. On the other hand, users may become anxious when others are watching them, thus disengaging from SSTs, as they may perceive that they are difficult to use [52]. Consequently, social anxiety is likely to be associated with users’ problem-based and emotion-based coping behaviors in SST contexts.

**Hypothesis** **1a.** *Elderly users’ social anxiety about SSTs is positively related to the problem-based coping behavior of seeking interaction.*

**Hypothesis** **1b.** *Elderly users’ social anxiety about SSTs is positively related to the problem-based coping behavior of observing others.*

**Hypothesis** **1c.** *Elderly users’ social anxiety about SSTs is positively related to the emotion-based coping behavior of behavioral disengagement.*

Older people often struggle to use digital technology, and feelings of helplessness may intensify from experiences of failure and frustration [53]. Helplessness is likely to occur when individuals perceive either a low possibility of achieving an objective or an incongruent experience [38]. Helplessness does not indicate a responsibility judgment, implying that it may not lead to aggressive responses [54]. Given individuals’ perceived lack of control in situations in which helplessness arises, helpless users are likely to seek assistance from employees. Moreover, helpless users may feel the need to control their frustrations by observing others’ actions and finishing a required process accordingly [55]. Behavioral disengagement is identified with the negative emotion of helplessness [43]. As behavioral disengagement may occur when individuals anticipate poor coping outcomes, helpless users may be behaviorally disengaged from SSTs. As negative experiences with SSTs often trigger feelings of helplessness [56], it may encourage problem-based and emotion-based coping behaviors.

**Hypothesis** **2a.** *Elderly users’ helplessness about using SSTs is positively related to the problem-based coping behavior of seeking interaction.*

**Hypothesis** **2b.** *Elderly users’ helplessness about using SSTs is positively related to the problem-based coping behavior of observing others.*

**Hypothesis** **2c.** *Elderly users’ helplessness about using SSTs is positively related to the emotion-based coping behavior of behavioral disengagement.*

### 2.6. Influences of Elderly-Specific Characteristics on Emotions and Behaviors

#### 2.6.1. Perceived Physical Condition (PPC)

As aging is a constant and highly complicated process, older people’s perceived physical condition may affect their interactions with technology [57]. Biological factors account for age-related declines in their physical abilities, including their speed in responding to technologies. Consequently, older people are often perceived as being “laggards” with regard to technology. The elderly experience declines in the speed of execution, mobility, and agility [58]. It is difficult for older adults to easily conduct duties that are physically demanding, involving swift movement and standing [59]. Another important consideration is the deterioration of eyesight, as most information presented by interfaces is visual [60].

Perceived physical condition (PPC) indicates perceptions of one’s physical struggles with hearing, vision, and movement in everyday life [61]. Physical decline may negatively affect older people’s experiences with SST, impeding them from making precise choices using the interface. Deterioration of physical abilities may make it difficult for older users to process transactions with SSTs, which can trigger negative emotional responses of social anxiety and helplessness.

**Hypothesis** **3a.** *PPC is positively related to social anxiety about SST.*

**Hypothesis** **3b.** *PPC is positively related to helplessness about SST.*

#### 2.6.2. Cognitive Losses

Age is typically accompanied by a deterioration in working memory, such as the ability to manage the information required to perform tasks related to ICT [62]. Indeed, older people perceive greater complexities in using technology due to their cognitive abilities. Awareness of age-related change (AARC) implies people’s perception that their performance, behavioral responses, and life experiences have been altered due to aging [63]. There are both cognitive gains and losses, the former of which refer to perceived improvements in knowledge, and the latter of which refer to perceived weaknesses in processing, memory, and mental ability [64]. The feeling of embarrassment associated with cognitive losses intensifies the social anxiety experienced in others’ presence. That is, users with weaker cognitive abilities are expected to exhibit higher social anxiety. We assume that cognitive decline is associated with feelings of helplessness, which are reinforced by negative experiences using SST [56]. Difficulties that arise with individuals’ decline in cognitive abilities may trigger emotions of helplessness when they are unable to proceed with the SST.

**Hypothesis** **4a.** *Cognitive losses are positively related to social anxiety about SST.*

**Hypothesis** **4b.** *Cognitive losses are positively related to helplessness about SST.*

### 2.7. Influences of Technical Characteristics on Emotions and Behaviors

#### 2.7.1. SST’s Reduction

Technical factors comprise the design and components that influence how older people work with technology [57]. Technical difficulty arises when the technology is perceived to be difficult, and users are afraid to learn about and use it [65]. The difficulty of the user interface hinders how individuals process the interface, which is generally tailored to younger people. The simplicity of reduction refers to the system that is designed in a way that only a minimum number of steps are necessary to carry out a task or transaction [66]. Reduction mainly refers to an application’s simplicity by virtue of which the technology is reduced to its fundamentals. Reduction can be applied to the aspects of technology or application design, which include functionality, navigational complexity, and interface complexity [67]. Particularly, the difficult manipulation of the SST interface pertains to controlling the touchscreen to complete the payment process, making it difficult for elderly users to use the SST and amplifying their social anxiety. Moreover, the interface’s complexity can create frustration, augmenting feelings of helplessness. Individuals may feel a perceived lack of control in situations in which helplessness arises, especially when older adults experience failures or are unable to proceed with transactions via the SST’s complex interface [54].

**Hypothesis** **5a.** *SST’s reduction is negatively related to social anxiety about SST.*

**Hypothesis** **5b.** *SST’s reduction is negatively related to helplessness about SST.*

#### 2.7.2. Perceived Ease of Use of SST

Perceived ease of use is defined as the degree to which SST is regarded as easy to operate [68]. Consumers’ attitudes toward an SST can be more positive when they perceive that the SST is useful or easy to use [69]. In this study, perceived ease of use indicates the hardware aspect of SST—the ease with which users can handle SST. SSTs that are neither usable nor accessible [70] may induce feelings of social anxiety in older people, especially in the presence of other consumers around the SST [71]. The difficulties and failures associated with using SST augment feelings of helplessness. Thus, we identified ease of use as the key technical construct affecting negative emotions.

**Hypothesis** **6a.** *Perceived ease of use is negatively related to social anxiety about SST.*

**Hypothesis** **6b.** *Perceived ease of use is negatively related to helplessness about SST.*

### 2.8. Influences of Situational Characteristics on Emotions and Behaviors

#### 2.8.1. Perceived Time Pressure

Perceived time pressure refers to how individuals understand time availability and sacrifice [72]. Time pressure takes place when choices must be made in a shorter time than is required to complete the task. It indicates the extent to which an individual recognizes oneself as lacking time concerning one’s everyday routines [73]. Perceived time pressure may be applied when older people use SST because they need to process the technology during their time on-site. Individuals who perceive that they are pressed for time tend to feel stressed when it is their turn to use the technology [74]. This type of pressure may be intensified in a situation where elderly users are unable to simplify their decision-making strategies for processing, augmenting their social anxiety. Moreover, as the transaction must be performed within a limited time span [51] with no direct assistance [75], the stress reinforces feelings of helplessness.

**Hypothesis** **7a.** *Perceived time pressure is positively related to social anxiety about SST.*

**Hypothesis** **7b.** *Perceived time pressure is positively related to helplessness about SST.*

#### 2.8.2. Perceived Crowding

In a self-service situation, the existence of many people waiting in line can exert pressure on users. Perceived crowding is a situational variable relevant to the on-site self-service environment. It refers to a subjective negative emotion that arises when an environment is considered to be concentrated [76]. The presence and number of other customers can arouse negative feelings and increase social anxiety (e.g., crowded retail shop; [77]). This is because crowding induces a certain level of stress and a lack of control within the environment, especially in retail settings [78]. Therefore, individuals often adjust their actions if others are watching them [79], which can augment social anxiety among elderly users. Additionally, others’ presence in line may reinforce feelings of helplessness or lack of control, with no assistance on the spot for immediate help [54].

**Hypothesis** **8a.** *Perceived crowding is positively related to social anxiety about SST.*

**Hypothesis** **8b.** *Perceived crowding is positively related to helplessness about SST.*

**Figure 1:**
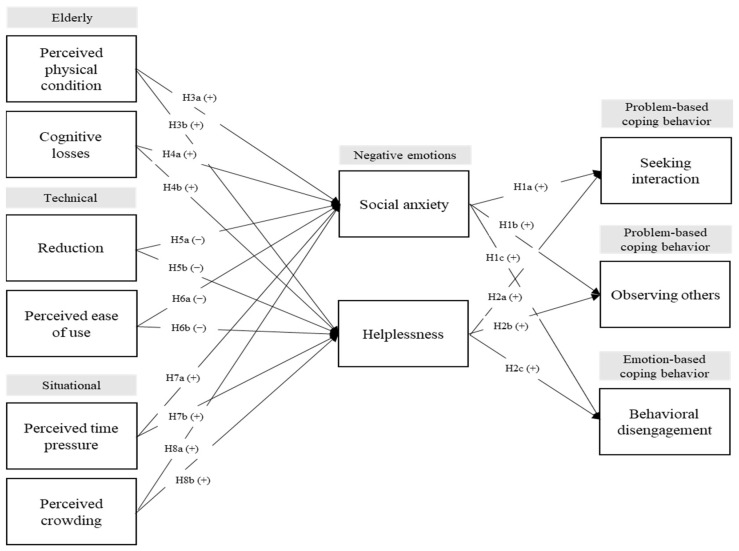
Research model.

Figure 1 summarizes our research model.

## 3. Methodology

### 3.1. Data Collection

Prior to conducting the off-site survey, a pilot study was employed from 5 December to 10 December 2021 to identify elderly users’ feelings toward SST. The pilot survey comprised open-ended questions about thoughts, emotions, and reactions pertaining to SST use. We aimed to identify the negative emotions associated with users’ coping responses. Based on the pilot study’s results and our literature review of emotional responses, we measured social anxiety and helplessness in the actual survey.

Data were collected off-site from 1 May to 24 May 2022, surveying South Koreans in their 60s and 70s with experience using SST in fast-food restaurants. Most prior studies consider individuals over 60 years old to be elderly [80,81]. We did not conduct an online survey because limiting the sample to elderly users who could effectively utilize online resources would produce biased results. Instead, we aimed to obtain direct and accurate responses from elderly individuals overall. Convenience sampling was appropriate in this context, selecting certain characteristics that are typically considered in the aging literature related to social and behavioral research [82,83,84]. Furthermore, several prior studies have successfully employed convenience sampling of elderly respondents [85,86,87]. The survey was conducted in places where elderly users are present, including elderly communities, senior centers, senior facilities, and elderly care worker facilities. We were unable to collect data in the fast-food restaurants themselves due to constraints posed by COVID-19. The content of the printed survey was written in Korean, and we initially checked the respondents’ ages and their experiences with SST in fast-food restaurants. Participants read text scenarios and viewed pictures of SST implemented in fast-food restaurants. We then measured users’ emotional responses and coping behavior. After excluding three unreliable responses, 100 valid questionnaires remained for the final analysis. As individuals in their 60s and 70s can be relatively difficult to reach [88,89,90], we deemed this sample of 100 respondents to be sufficient. A sample size of 100 is also considered adequate for partial least squares structural equation modeling (PLS-SEM; [91]). The respondents’ demographic factors are shown in Table 1.

### 3.2. Measures

The survey consisted of three main sections—measuring personal, technical, and situational factors, negative emotions, and subsequent coping behaviors, as well as demographic items. The survey consisted of items adapted from prior research, measured with a 5-point Likert scale ranging from 1 (“strongly disagree”) to 5 (“strongly agree”). The constructs of perceived physical condition and loss in cognition were adopted from prior research [61,64]. We also adopted measures of reduction, modifying the variable to fit the SST context [67]. We adopted the perceived ease of use construct from prior research [68]. Perceived time pressure and social pressure were also adapted from prior research [51,79]. The problem-based coping behavior of the need for interaction was adapted from a prior study [43]. Observing others was adopted from a previous study [55] but modified by the researcher. The emotion-based coping strategy of behavioral disengagement was adopted from prior research [43]. We also included items assessing the frequency of credit card use and familiarity with mobile payment.

### 3.3. Measurement Model

PLS-SEM using SmartPLS 3.0 was employed to investigate the research model and to test and estimate causal relations. PLS-SEM is an applicable method to use with small- to medium-sized samples [91]. This modeling method is suitable for assessing high predictive models and for theory building phases of exploratory research in situations where the research contexts have little theoretical background [92]. Given the small sample size and scant research on elderly-specific or SST-specific factors that may influence individuals’ response to SSTs, PLS-SEM was employed.

In measurement model estimation, the research model must satisfy criteria for reliability and validity. First, we judged the reliability of each item to be appropriate because each factor loading exceeded 0.7. The general criteria for constructs’ reliability include a Cronbach’s α exceeding 0.7 and composite reliability (CR) exceeding 0.8 [93]. We further assessed the measurement model’s reliability and validity by examining the CR and average variance extracted (AVE). The AVE value for every construct exceeded the threshold of 0.5, indicating sufficient convergent validity (Table 2). To establish discriminant validity, we examined the square root of the AVE for each latent variable [94]. The obtained values were higher than those for other correlations among the latent variables (Table 3). Thus, we established discriminant validity in our data. As PLS-SEM was used in the analysis, the values for the heterotrait-monotrait (HTMT) ratio were reported, which is an alternative index of discriminant validity. All values were below the recommended value of 0.85 (Table 4).

## 4. Results

**Figure 2:**
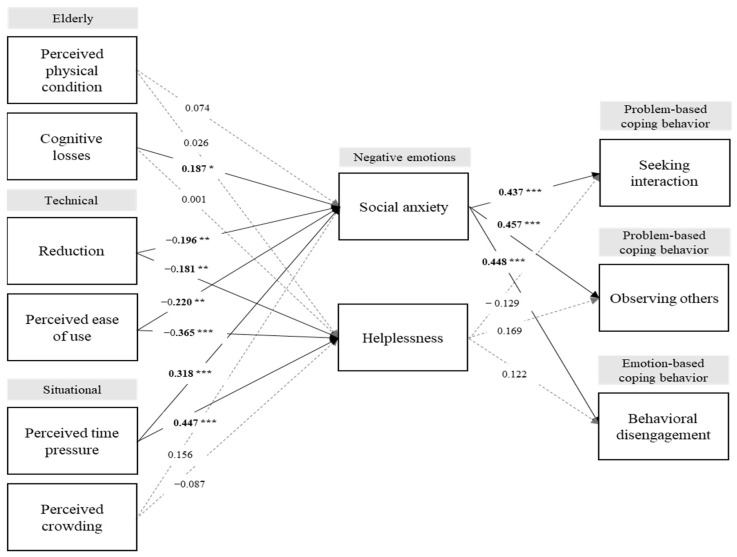
Research model test results; * *p* < 0.05, ** *p* < 0.01, *** *p* < 0.001.

The structural model employed 5000 bootstrap subsamples to assess the level of significance for the structural paths. We examined the relationship between emotional responses and coping strategies. Social anxiety was significantly related to seeking interaction (β = 0.437 ***, *t* = 3.496, *p* < 0.001), observing others (β = 0.457 ***, *t* = 4.507, *p* < 0.001), and behavioral disengagement (β = 0.448 ***, *t* = 4.505, *p* < 0.001). However, helplessness had no significant relationship with the three coping strategies. Therefore, H1a, H1b, and H1c were supported, while H2, H2b, and H2c were not supported. Furthermore, in terms of the relationships between the determinants of users’ emotional responses toward SST and coping behaviors, perceived physical condition had no significant relationship with social anxiety or helplessness; thus, H3a and H3b were not supported. Cognitive losses were significantly related to social anxiety (β = 0.187 *, *t* = 2.003, *p* < 0.05) but not to helplessness, supporting H4a but not H4b. Supporting H5 and H6, the technical factors of reduction and perceived ease of use were significantly related to the negative emotions of helplessness and social anxiety in response to SST: reduction and social anxiety (β = −0.196, *t* = 2.390, *p* < 0.01); reduction and helplessness (β = −0.181, *t* = 2.164, *p* < 0.01); perceived ease of use and social anxiety (β = −0.220, *t* = 2.287, *p* < 0.01); and perceived ease of use and helplessness (β = −0.365, *t* = 3.722, *p* < 0.001). Moreover, H7 and H8 predicted that the situational factors of perceived time pressure and perceived crowding would be significantly related to users’ social anxiety and helplessness. The results showed that perceived time pressure was significantly related to social anxiety (β = 0.318, *t* = 3.815, *p* < 0.001) and helplessness (β = 0.447, *t* = 4.031, *p* < 0.001), supporting H7a and H7b. On the other hand, perceived crowding was not significantly related to either social anxiety or helplessness; thus, H8a and H8b were not supported. Table 5 and Figure 2 summarize the results of hypothesis testing.

## 5. Discussion

### 5.1. Key Findings

This study had several key findings. The results of the PLS-SEM analysis revealed that social anxiety is a crucial determinant of users’ problem-solving coping strategies, which include seeking interaction with employees and observing others using the SST. Users who feel socially anxious experience discomfort in specific social situations where they may be scrutinized by others. To cope with such uncomfortable situations, users tend to seek assistance from employees. Social anxiety is also associated with users’ observational learning, in which they imitate others’ behavior to complete transactions using SST. These findings align with prior studies showing that a positive coping strategy protects against social anxiety [95]. The results also showed that social anxiety is associated with users’ emotional coping response to behavioral disengagement. Accordingly, as suggested by prior literature, social anxiety appears to elicit adaptive behaviors to reduce a situation’s risk level [96]. With social pressures, users are likely to avoid using new and unfamiliar technology [97], leading them to disengage from the SST. These findings are relevant to how older people particularly tend to be more sensitive to how they appear to others, considering social status and self-presentation to be important. The emphasis on one’s appearance to others is strongly reflected in Asian culture [98]. The findings further explain how social anxiety is associated with cultural norms and values across countries. South Korea is considered to have a collectivistic culture, and prior research reported that collectivistic regions may exhibit higher social anxiety and positive attitudes toward behaviors that are socially avoidant [99].

Furthermore, contradicting prior research showing that helplessness may elicit emotion-based coping [100], helplessness had no significant relationship with either problem-based or emotion-based coping behavior in this study. These results may imply that helpless individuals experiencing a severe lack of control that may still not be sufficient to induce coping behavior are frequently reinforced by the failed experiences that induce frustration. Moreover, empirical results suggest that helplessness alone may not be adequate to induce active coping in a situation [54]. This makes a nonsignificant relationship between helplessness and coping seem reasonable.

In terms of the relationship between elderly-specific characteristics and negative emotional responses, while perceived physical condition did not influence social anxiety or helplessness about SST, cognitive losses increased social anxiety among elderly users. Despite prior research showing that older people’s deteriorating physical abilities impede them from using technology [60], the current findings revealed that physiological conditions did not affect negative emotions. One possible explanation is that elderly people do not experience such intense physical difficulties. On the other hand, cognitive losses were positively associated with social anxiety but not helplessness. The findings suggest that users who face difficulties due to reduced cognitive abilities are more likely to experience social anxiety when facing social pressures. Older people may feel more socially anxious due to embarrassment associated with declining cognitive abilities and short-term memory loss [101].

Regarding technical factors, SST’s reduction was negatively related to social anxiety and helplessness. The findings showed that the higher the reduction of the SST interface, the lower the social anxiety individuals experienced. A simple SST user interface is likely to make individuals feel in control and less helpless. Moreover, perceived ease of use was negatively related to social anxiety and helplessness. SST that is perceived as easy to use may mitigate negative emotional responses. Subsequently, users may become more frustrated with a technological system that they perceive as challenging to use [102].

Perceived time pressure was the strongest predictor of social anxiety and helplessness, while perceived crowding did not influence either negative emotion. When individuals perceive themselves as lacking time in their daily tasks of living, this can induce stress [74], which may strengthen the negative influence of social anxiety. Moreover, individuals who perceive that they are pressed for time when completing a transaction may find themselves feeling helpless. This aligns with prior research suggesting that situational factors, such as time pressure, may affect reactions to SST [103]. In contrast, perceived crowding did not influence helplessness. A possible explanation is that the stress generated by perceived crowding is not strong enough to reinforce the negative effect of social anxiety or helplessness on older people’s use of SST.

### 5.2. Implications for Theory and Practice

This study has several important implications for theory and practice. SSTs will likely become commonplace in the near future. Accordingly, older people will continue to experience challenges in using SST due to their relatively lower levels of digital literacy. However, prior studies have concentrated on the general factors that lead to older people’s use of SST, with less research being conducted on elderly users’ actual emotional and behavioral responses to SST. To address this gap, this study analyzed the relationships among individual factors, negative emotional responses, and coping behavior. The current findings contribute to a richer understanding of SST from older people’s perspective.

This study confirms the significant roles of elderly-specific factors, technical factors, and situational factors in the negative emotions experienced by older people in response to SSTs, as well as their subsequent coping behaviors. We applied elderly-specific factors that could reflect the complicated situation of elderly SST users, considering them in terms of physical and cognitive abilities, especially concerning cognitive losses. The results showed that cognitive losses influenced elderly users’ negative emotional response and their coping strategies, implying that the SST’s interface should be designed with settings that cater to different situations faced while using the kiosk, which can evaluate and compensate for elderly users’ declines in cognitive abilities. Moreover, this study’s examination of the technical factor, reduction of SST, extends the concept of simplicity, which is critical in human–computer interaction research.

This study’s findings have implications for SST service providers, fast-food restaurants, academia, and government in terms of taking a more integrative approach to the implementation of self-service kiosks. These results suggest that service providers should create a clear and simple kiosk interface with minimal transaction steps, clear and simple menu items presented in larger fonts, and accessible color schemes. An easy-to-use SST would increase accessibility. For the government, the implementation of nationwide digital inclusion policies is critical to protecting older people’s interests, helping to bridge the digital divide. Currently, the National Assembly is discussing the enactment of the ‘Digital Inclusion Act’ that focuses on the establishment of a digital inclusion committee, nurturing of professionals, and measures for digital access for older adults and those with disabilities. Efforts for comprehensiveness in public policy regulations at the national, regional, and local levels are necessary to ensure that digital inclusion-related policies facilitate elderly people’s technology use. Furthermore, a comprehensive approach is needed for appropriate e-government policies and e-service tools to help reduce the digital divide. For example, nationwide ‘kiosk accessibility standards’ that center around a simple and usable interface should be developed. The SST should be accessible to all individuals. Elderly users may believe that they are inferior ICT users, inducing negative emotions and subsequently affecting their quality of life. Therefore, it is critical to combat users’ feelings of vulnerability regarding technology use.

The findings also provide insights into the opportunities and challenges for academic institutions when resourcing and implementing digital literacy programs. Digital literacy is a necessary skill for leveraging available digital technology to meet informational needs [104]. Digital education for elderly people has become a necessity, as South Korea experiences digitization in various sectors. Specifically, government-led education programs and digital literacy interventions should be coordinated at the community level through the joint efforts of diverse organizations. Private and public institutions, community centers, and extension colleges with digital literacy programs for older people are appropriate for them to learn how to use SST. Therefore, collaboration among the government, the private sector, academia, and organizations will produce inclusive digitalization outcomes—both publicly and privately—which will be critical in addressing the digital divide.

Lastly, the study provides insights into alternative coping strategies that elderly users can follow when using SST. For users who need human interaction, an employee can stand beside the SST and aid them when difficulties emerge. For users who learn how to use technologies by observing others, it is necessary to present simple and well-explained instructions on the SST interface that can facilitate the independent use of the technology. For users who disengage from using SSTs, further education or promotional programs are needed to reduce their negative emotions. As users have stated that facing unfamiliar kiosks induces uncomfortable feelings, increasing familiarity with technology will be critical to increasing users’ confidence.

## 6. Conclusions

This study specifically explored SST implementation in fast-food restaurants with respect to elderly users’ responses. With ongoing COVID-19, digital transformation will play a vital role in individuals’ lives. It has been shown that older adults are the most disadvantaged group with respect to technology use. Despite such problems, most prior research has explored the general factors that influence elderly users’ intentions to use SST. To address this research gap, this study examined the relationships among personal, technical, and situational factors, negative emotions, and coping behaviors among elderly SST users.

This study is not without its limitations. The study identified the antecedents of older people’s emotional and behavioral responses to SST. However, the factors suggested in this study may not comprehensively describe elderly users’ rich experiences with SST. In this sense, it is necessary to investigate how individuals’ digital literacy, psychological, or personality factors may influence their responses. Further research is required to verify new constructs that can fully explain the research model in the context of elderly SST users.

Additionally, this research took place in South Korea, a leading country in ICT development but a country experiencing a digital technological divide, especially for older people. Although the fundamental constructs and the process can be applicable to other countries, it may be difficult to generalize the findings to other regions. Furthermore, this study produced meaningful results in that social anxiety was a major predictor of coping responses among elderly South Korean SST users. As social anxiety is presumably associated with cultural norms and values across countries [105], it will be meaningful to conduct cross-cultural research examining older people’s usage of SST in individualistic versus collectivistic cultures.

This study employed the convenience sampling method, in which the researchers visited a limited number of locations where surveys could be filled out during COVID-19, implying that there may be selection bias in the results. To increase the generalizability of the findings, future research should employ larger-scale surveys exploring the socio-demographic differences within the older population, as well as comparing different age groups’ use of SST. Lastly, this study considered one specific setting for SST usage: fast-food restaurants. SST has been implemented in various contexts, such as hospitals, banks, civil services, and bus terminals, becoming an integral part of individuals’ everyday lives. Accordingly, future studies should examine whether there are significant differences in individual factors, emotions, and coping behaviors in different SST settings.

## Figures and Tables

**Table 1:** Demographic Information.

Measures		Frequency	Percent
Gender	Male	52	46.4
	Female	48	42.9
Age	60–64	39	34.8
	65–69	42	37.5
	70–74	13	11.6
	75–79	6	5.4
Education	Elementary school	1	1.0
	Middle school	3	3.0
	High school	17	17.0
	College	19	19.0
	University	40	40.0
	Graduate school	20	20.0
Kiosk usage in	1–2 times in a month	72	64.3
fast-food restaurants	3–4 times in a month	15	13.4
	5–6 times in a month	3	2.7
	>7 times in a month	2	1.8
	Once in a 2–3 month	3	2.7
	Once in 6 months	5	4.5
Time spent for	1–3 min	57	50.9
kiosk completion	4–6 min	36	32.1
	7–9 min	2	1.8
	10–15 min	5	4.5

**Table 2:** Construct reliability.

Construct	No. of Items	Item Loading	Cronbach’s Alpha	CR	AVE
Perceived physical condition	4	0.894–0.945	0.940	0.957	0.848
Cognitive losses	4	0.826–0.916	0.910	0.937	0.789
Reduction	4	0.911–0.947	0.953	0.966	0.876
Perceived ease of use	4	0.839–0.897	0.894	0.927	0.760
Perceived time pressure	4	0.801–0.895	0.880	0.917	0.736
Perceived crowding	4	0.909–0.953	0.948	0.962	0.864
Social anxiety	4	0.901–0.957	0.945	0.960	0.859
Helplessness	4	0.939–0.970	0.972	0.979	0.921
Seeking interaction	4	0.655–0.803	0.753	0.826	0.544
Observing others	4	0.838–0.970	0.942	0.958	0.851
Behavioral disengagement	4	0.816–0.889	0.871	0.911	0.718

**Table 3:** Discriminant validity (Fornell and Larcker, 2015; [94]).

Constructs	PPC	LIC	R	PEOU	PTP	PC	SA	H	NI	OO	BD
Perceived physical condition	0.921										
Cognitive losses	0.663	0.888									
Reduction	−0.096	−0.055	0.936								
Perceived ease of use	−0.214	−0.200	0.457	0.872							
Perceived time pressure	0.307	0.199	−0.357	−0.464	0.858						
Perceived crowding	0.181	0.232	−0.372	−0.168	0.461	0.930					
Social anxiety	0.390	0.390	−0.486	−0.536	0.621	0.469	0.927				
Helplessness	0.244	0.170	−0.477	−0.646	0.649	0.253	0.586	0.960			
Seeking interaction	0.138	0.134	−0.358	−0.460	0.327	0.273	0.517	0.396	0.738		
Observing others	0.219	0.270	−0.130	−0.219	0.236	0.114	0.356	0.124	0.375	0.922	
Behavioral disengagement	0.195	0.269	−0.294	−0.480	0.460	0.210	0.520	0.388	0.332	0.171	0.847

**Table 4:** Discriminant validity (Heterotrait-Monotrait (HTMT)).

Constructs	PPC	LIC	R	PEOU	PTP	PC	SA	H	NI	OO	BD
Perceived physical condition											
Cognitive losses	0.218										
Reduction	0.100	0.072									
Perceived ease of use	0.233	0.218	0.490								
Perceived time pressure	0.334	0.218	0.386	0.518							
Perceived crowding	0.192	0.248	0.388	0.179	0.497						
Social anxiety	0.41	0.418	0.508	0.584	0.677	0.493					
Helplessness	0.254	0.179	0.492	0.690	0.704	0.260	0.612				
Seeking interaction	0.137	0.139	0.387	0.509	0.342	0.252	0.499	0.446			
Observing others	0.230	0.298	0.130	0.227	0.243	0.120	0.358	0.120	0.435		
Behavioral disengagement	0.215	0.286	0.309	0.540	0.504	0.229	0.558	0.402	0.327	0.175	

**Table 5:** Hypothesis testing results.

Hypothesis	β	*t*	Results
H1a: Social anxiety → Seeking interaction	0.437 ***	3.496	Supported
H1b: Social anxiety → Observing others	0.457 ***	4.507	Supported
H1c: Social anxiety → Behavioral disengagement	0.448 ***	4.505	Supported
H2a: Helplessness → Seeking interaction	0.129	0.861	Not supported
H2b: Helplessness → Observing others	−0.169	1.350	Not supported
H2c: Helplessness → Behavioral disengagement	00.122	0.890	Not supported
H3a: Perceived physical condition → Social anxiety	.074	0.793	Not supported
H3b: Perceived physical condition → Helplessness	0.026	0.257	Not supported
H4a: Cognitive losses → Social anxiety	0.187 *	2.003	Supported
H4b: Cognitive losses → Helplessness	0.001	0.014	Not supported
H5a: Reduction → Social anxiety	−0.196 **	2.390	Supported
H5b: Reduction → Helplessness	−0.181 **	2.164	Supported
H6a: Perceived ease of use → Social anxiety	−0.220 **	2.287	Supported
H6b: Perceived ease of use → Helplessness	−0.365 ***	3.722	Supported
H7a: Perceived time pressure → Social anxiety	0.318 ***	3.815	Supported
H7b: Perceived time pressure → Helplessness	0.447 ***	4.031	Supported
H8a: Perceived crowding → Social anxiety	0.156	1.858	Not supported
H8b: Perceived crowding → Helplessness	−0.087	0.851	Not supported

*Note*. * *p* < 0.05, ** *p* < 0.01, *** *p* < 0.001.

## Data Availability

The data are available from the corresponding author upon reasonable request.
